# The Need for an Economically Feasible Nursing Home Staffing Regulation: Evaluating an Acuity-Based Nursing Staff Benchmark

**DOI:** 10.1093/geroni/igac017

**Published:** 2022-03-21

**Authors:** John R Bowblis

**Affiliations:** Department of Economics and Scripps Gerontology Center, Miami University, Oxford, Ohio, USA

**Keywords:** Nursing homes, Nursing staff regulations, 1995/97 Staff Time Measurement studies, Sufficient nursing staff

## Abstract

**Background and Objectives:**

Despite concerns about the adequacy of nursing home (NH) staffing, the federal agency responsible for NH certification and regulation has never adopted an explicit quantitative nursing staff standard. A prior study has proposed a benchmark for this purpose based on the 1995/97 Staff Time Measurement (STM) studies. This article aims to assess the extent to which NHs staff to this proposed STM benchmark, the extent to which regulators already implicitly apply the STM benchmark, and compute the additional operating expenses NHs would incur to adhere to the STM benchmark.

**Research Design and Methods:**

Using NH Compare Archive data, the STM benchmark was compared to staffing levels reported by the facility and whether NHs received a nursing staff deficiency. Using financial information from Medicare Cost Reports, the additional annual operating expenses required to staff to the STM benchmark were calculated for each state and nationwide.

**Results:**

The vast majority of NHs did not staff to the STM benchmark; 80.2% for registered nurses and 60.0% for total nursing staff. Deficiency patterns showed that NH regulators were not using the STM benchmark to determine sufficiency of nursing staff. Implementing the STM benchmark as a regulatory standard would increase operating expenses for 59.1% of NHs, at an average annual cost of half-million dollars per facility. The nationwide increase in operating expense is estimated to be at least $4.9 billion per year.

**Discussion and Implications:**

Without clear guidance on the staffing level needed to be sufficiently staffed, most NHs are subject to a community standard of care, which some have argued could be associated with suboptimal staffing levels. Implementing an acuity-based benchmark could result in improved staffing levels but also comes with significant economic costs. The STM benchmark is not economically feasible at current Medicare and Medicaid reimbursement levels.

**Translational Significance:** Policymakers could provide better guidance on when a nursing home (NH) is sufficiently staffed. This study examines whether a proposed acuity-adjusted benchmark for staffing levels based on the 1995/97 Staff Time Measurement studies is economically feasible. The benchmark is not associated with current regulatory standards but is likely to result in higher nursing staff levels at many NHs. However, implementing this type of benchmark has economic costs that make the proposal economically infeasible without revenue offsets, most likely from the Medicaid program.

Nursing staff plays a vital role in caring for nursing home (NH) residents, as nursing staff assess clinical needs, assist with activities of daily living, and provide other care and services. Federal NH regulations require NHs to have “sufficient nursing staff” to care for residents (§483.35[a]). This is in addition to having a licensed nurse (i.e., registered nurse [RN] or licensed practical nurse [LPN]) on duty at all times with at least eight consecutive hours being staffed by a RN (§483.35[b]). Federal regulations do not currently provide a specific quantitative definition or benchmark to determine the nursing staff level associated with being sufficiently staffed (Centers for Medicare and Medicaid Services [CMS], 2017). The absence of a clear CMS benchmark necessitates NHs to rely on other devices to determine if they are sufficiently staffed. This device is often the legal concept connected to medical malpractice and negligence lawsuits of the “community standard of care.” The legal definition of community standard of care is specific to each state but is conceptualized as what is “usual and customary.” Under the community standard of care, the market determines when a NH is sufficiently staffed.

In an attempt to provide guidelines and assure there are a minimum number of nurses and nurse aides per resident, the majority of states have minimum nursing staff regulations. The staffing levels required by these state regulations vary significantly (Mueller et al., 2006). More importantly, research shows that when states implement brand new minimum nursing staff regulations or increase the mandated minimum required by existing regulations, nursing staff levels at most NHs converge towards slightly above the minimum (Bowblis, 2011; Bowblis & Ghattas, 2017; Park & Stearns, 2009). In other words, minimum nursing staff regulations often cause the community standard of care to become the minimum staffing level required by state regulations. Some state regulatory codes use language that reinforces these minimum requirements as the community standard of care. This is illustrated in the California regulatory code (22 CCR §72329.2), which states that NHs “shall employ *sufficient nursing staff* to provide a minimum of 3.5 direct care services hours per patient day” (emphasis added).

While state minimum staffing regulations are the de facto community standard of care to be sufficiently staffed in most states, some NH advocates question whether the minimums imposed by state regulations are high enough to provide adequate care (Harrington et al., 2020). In addition, many state minimum staffing regulations only apply a single minimum staff-to-resident ratio that is applicable to all NHs regardless of size, case-mix, or revenue streams, despite these varying significantly across facilities. For these and other reasons, a small number of NH advocates have called for CMS and state regulators to develop a quantitative benchmark for when a NH is sufficiently staffed.

Recently, Harrington et al. (2020) recommended just such a benchmark that accounts for the acuity of residents. They argue their proposed benchmark is a “guide for determining whether a facility has adequate and appropriate nurse staffing.” Their proposal starts with using resident-level assessments from the Minimum Data Set (MDS). These MDS assessments collect health conditions and services provided to each NH resident at least every 90 days. Most MDS assessments include a Resource Utilization Group (RUG) billing code. Harrington et al. argue this RUG billing code is a proxy for each resident’s acuity. Next, each RUG billing code is assigned a nursing staff time. Theoretically, this nursing staff time should be the amount of nursing staff time needed when caring for a resident with that particular RUG billing code. Finally, the nursing staff time for each resident is aggregated to the facility-level to obtain the average nursing staff time per resident. According to Harrington et al. (2020), the average nursing staffing time per resident is the staffing level benchmark that would determine whether “nursing staffing in a nursing home is sufficient.”

While there are multiple assumptions associated with this proposal, two key components are: (a) the RUG billing codes from infrequent MDS assessments accurately reflect a resident’s acuity and preferences, and (b) the nursing staff times assigned to each RUG billing code accurately reflects the nursing staff time associated with appropriately caring for that resident. CMS has conducted 1995/97 Staff Time Measurement (STM) studies (henceforth “STM studies”) and the Staff Time and Resource Intensity Verification (STRIVE) study that both assign nursing staff times to each RUG billing code (see Author Note 1). However, these studies were designed to determine reimbursement rates for Medicare and were not designed to calculate the nursing staff times associated with being sufficiently staffed.

The suggested benchmark for sufficient staffing levels proposed by Harrington et al. (2020) uses the STM studies for nursing staff times (see Author Note 2). Even though the STM studies were not designed to determine if a NH was sufficiently staffed, one of the reasons the authors justified the use of the STM studies to construct the benchmark is the nursing staff times associated with the STM studies are associated with higher staffing levels than the STRIVE study. Increasing staffing levels has merit in potentially improving the quality of care as there is a consensus that higher nursing staff levels are generally associated with higher quality (Bowblis, 2011; Chen & Grabowski, 2015; Park & Stearns, 2009; Tong, 2011). However, the full consequences of using any staffing benchmark needs to be known before policymakers should consider implementing them.

The key limitation of the benchmark Harrington et al. (2020) propose is the absence of empirical tests for feasibility. If a staffing benchmark is adopted by regulators and NHs, it must meet certain requirements. For example, regulators must easily verify compliance. Additionally, it is essential to understand whether the benchmark would result in staffing levels that are higher or lower than how NHs are already operating. A benchmark that leads to lower staffing levels would likely not result in improved quality, whereas a method that increases staffing levels could result in better quality. Moreover, even though the benchmark is not written into the regulatory code, regulators may already utilize the benchmark. Therefore, it is important to understand whether or not regulators issue deficiencies for not being sufficiently staffed based on the difference in how NHs are staffed relative to the benchmark. Finally, it is important to determine whether the benchmark is economically feasible. In other words, feasibility requires that a NH could staff to the benchmark at a reasonable cost from currently available revenue.

The purpose of this article was to make some of these assessments for the benchmark proposed by Harrington et al. (2020) that uses nursing staff times from the STM studies, henceforth the “STM benchmark.” The first objective (Objective 1) was to understand the relationship between the STM benchmark, staffing levels, and nursing staff deficiencies when NHs underwent their annual recertification survey. Using Nursing Home Compare (NHC) Archive data from 2013 to 2018, the staffing levels reported by the facility during recertification surveys were compared to STM benchmark staffing levels. NHs were classified as having nursing staff levels above or below the STM benchmark to determine if NHs already staff to the benchmark. Next, it was determined if regulators were already implicitly applying the STM benchmark. More specifically, it was determined whether NHs staffed above and below the benchmark were issued a deficiency for not being in substantial compliance with federal nursing staff regulations.

The second objective (Objective 2) utilized financial data from 2017 Medicare Cost Reports (MCR) and the STM benchmarks from the NHC Archive data to understand the economic feasibility of the STM benchmark. If a NH staffed below the STM benchmark, the NH would be required to hire additional nursing staff to attain the STM benchmark. This would increase the NH’s operating expense. Hence, the economic feasibility of the STM benchmark was assessed by calculating the additional operating expense a NH would incur on an annual basis to staff to the STM benchmark. The proportion of NHs that would need to increase annual operating expenses, the dollar amount of those increased annual operating expenses per NH, and whether a NH could profitability staff to the STM benchmark were calculated. Additionally, the annual increase in operating expenses at the state and national level were calculated to understand whether Medicaid or Medicare would need to increase funding to NHs to staff to the STM benchmark.

## Research Design and Methods

### Data Sources

To understand the relationship between the STM benchmark, staffing levels, and nursing staff deficiencies when NHs underwent their annual recertification survey, Objective 1 utilized facility-level information obtained from the NHC Archive data. The NHC Archive data contained historical information reported on the NHC website, which was rebranded as the Care Compare website in late 2020. The data included when the facility was first certified, whether the NH was freestanding or hospital-based, the staffing levels reported by the facility associated with the recertification survey, a measure of the STM benchmark nursing staff levels associated with the recertification survey until March 2018, and the deficiencies the NH received.

The NHC Archive data utilized for Objective 1 consisted of information drawn from annual recertification surveys completed from January 1, 2014 to March 31, 2018. All NHs were required to undergo recertification surveys every 9–15 months (12 months on average) in order to assure NHs were substantially complying with regulations. This 51-month period captured the most recertification surveys available that also contain measures of the STM benchmark in the NHC Archive data (see Author Note 3). Objective 1 had a unit of observation of a recertification survey. The analytic sample consisted of 54,886 recertification surveys from freestanding NHs in the continental United States that were first certified prior to 2013.

To understand the economic feasibility of the STM benchmarks, Objective 2 utilized the STM benchmarks from NHC Archive data and financial information. The financial information for freestanding NHs were collected by CMS on the CMS-2540-10, which is more formally known as an MCR. The MCRs contained facility census, payer-mix, nursing staff levels and expenditures, and other financial information such as profitability. The sample was restricted to NHs that did not change ownership, had a fiscal year-end date of 2017, and a full-year cost report (i.e., reporting period equal to a year). These restrictions were made because nursing wages were generally increasing over the 2010s, ownership changes may lead to disruptions, and this study period reflects the most recent calendar year in which the STM benchmarks were available in the NHC Archive data. STM benchmarks for each facility were obtained from the NHC Archive data for the 12-month period that best matched each NH’s fiscal year. After excluding outliers, the unit of observation for Objective 2 reflected a NH in fiscal year 2017. The final analytic sample included 12,117 NHs. Outliers were identified as all observations that contained wage rates for each type of nursing staff that were in the 1% tails and MCRs that reported aberrant nursing staff levels as per CMS (2019a).

### Measures

#### Actual nursing staff levels

NHs employ three types of nursing staff: RNs, LPNs, and certified nurse aides (CNAs). For each of these types of nursing staff, staffing levels were measured in hours per resident day (HPRD), defined as the total number of hours associated with the staff type divided by the corresponding resident census. The MCRs separated nursing staff with administrative duties from those providing direct care whereas the NHC Archive data do not. However, the STM benchmark, which is based on the STM studies included a “resident-specific” and “nonresident-specific” time component. The “nonresident specific component included all-time not directly related to a specific resident, such as meetings, *nursing unit administration*, and staff meal times” (Federal Register, 1999, p. 41,663; emphasis added). To be consistent with how nursing staff is defined for the STM benchmark, this study utilized nursing staff levels that included those providing direct care and administrative services.

For Objective 1, the nursing staff level reported by the facility during the recertification survey was utilized as per the NHC Archive data (see Author Note 4). For Objective 2, nursing staff levels were obtained from the MCRs. The MCRs provided the number of hours paid to nursing staff. These hours were obtained from Worksheet S-3 Part V, Column 4 for nursing staff directly employed by the facility and contracted labor. Some NHs reported administrative RN time, such as the Director of Nursing, on a different worksheet. RN hours also included the number of hours of administrative RN time reported on Worksheet S-3 Part III, Line 9, Column 4. The facility’s census was obtained from Worksheet S-3 Part I, Line 1, Column 7.

#### STM benchmark nursing staff levels

The STM benchmark was sourced from the “case-mix hours” available in the NHC Archive data. “Case-mix hours,” which the NHC Archive data reported as staffing levels in HPRD, were calculated by CMS from the STM studies as part of determining a NH’s staffing star rating from 2009 to March 2018 (see Author Note 5). During this period, the CMS updated the staffing star rating, and “case-mix hours” every time CMS received a new recertification survey. Because Objective 1 used a recertification survey as a unit of observation, STM benchmark was defined as the “case-mix hours” associated with each recertification survey per CMS (2015). The unit of observation for Objective 2 reflected a NH fiscal year and the actual staffing levels reflect average staffing for a one-year period. To account for this time dimension, the STM benchmark utilized was the average “case-mix hours” reported in the NHC Archive data for the first month of each quarter reflected in the NH’s fiscal year.

#### Nursing staff deficiencies

Two nursing staff deficiencies were examined as part of Objective 1: “sufficient nursing staff” and “registered nurses.” Because CMS revised federal NH regulations in November of 2017, CMS’ mapping algorithm assured that the same regulation was applied. Deficiencies for “sufficient nursing staff” were identified by F-tags 353 and 725. Deficiencies for “registered nurses” were identified by F-tags 354 and 726.

#### Additional operating expenses staffing to STM benchmarks

As outlined in Objective 2, NHs could incur additional expenses in order to staff to the STM benchmark. To calculate these additional operating expenses, a multiple-step, facility-specific process was utilized. For each nursing staff type (e.g., RNs), the first step was to calculate the difference in the facility’s actual staffing level and STM benchmark staffing level. This provided the number of hours per resident the facility staffed above or below the STM benchmark on an average day in the year. The second step determined the total number of hours the facility is staffed above or below the STM benchmark for the entire year. The calculation multiplied the difference in actual and benchmark staffing levels obtained in the first step by the facility’s annual census (i.e., total resident days from MCR Worksheet S-3 Part I, Line 1, Column 7). Third, these hours were monetized by multiplying the total number of hours by the average wage rates (including fringe) for the corresponding nursing staff type. The average wage rate for each NH was obtained by taking the total expenditures on nursing staff (Worksheet S-3 Part III and V, column 3) divided by the number of hours reported in the MCR. Finally, the monetized values for each nursing staff type were summed across the three types of nursing staff to obtain the additional operating expenses associated with staffing to the STM benchmark. Positive values indicated that the NH would need to increase operating expenses and negative values indicated that facility could change its current nursing staff mix to the STM benchmark without incurring additional operating expenses.

### Analysis

To determine whether NHs staffed above or below the STM benchmark, Objective 1 calculated for each recertification survey the difference between the actual and STM benchmark staffing levels. These differences were reported in histograms for all nursing staff types and for total nursing staff (=RNs + LPNs + CNAs). Next, each recertification survey was classified into those for which the actual staffing levels were above the STM benchmark (“Yes”) and those for which the opposite was true (“No”). The proportion of recertification surveys that were classified into each category was calculated. Among the recertification surveys classified as “No,” the average difference in the actual and STM benchmark staffing levels was calculated. Objective 1 also determined whether regulators were already implicitly applying the STM benchmark by calculating the proportion of recertification surveys that resulted in a nursing staff deficiency. These proportions were calculated for surveys for which the NH was staffed above and below the STM benchmark staffing level.

To understand the economic feasibility of the STM benchmark, Objective 2 started with determining the additional operating expenses associated with staffing to the STM benchmark for each facility. These annual operating expenses were used to calculate the proportion of NHs that would be subject to increased operating expenses by staffing to the STM benchmark. Additionally, for NHs subject to increased operating expenses, the amount of the increased expenses for each facility were calculated, and summarized as means and medians. Finally, to assess the effect of staffing to the STM benchmark on profitability, the proportion of NHs that were unprofitable staffing to the STM benchmark were calculated. To determine whether a NH could profitably staff to the STM benchmark, additional operating expenses were subtracted from the net income (Worksheet G-3, Line 5). If the result was a positive value, the NH could have profitably staffed to the STM benchmark; otherwise, the NH was not profitable. To understand the scope of the additional operating expense across states, the proportion of NHs with increased annual operating expenses to staff to the STM benchmark were calculated for each state in the continental United States. For NHs that experienced increased operating expenses, the average annual per resident and per facility increase in operating expenses were calculated. The total increased operating expenses were aggregated to the state level. States were also ranked based on the highest total increase in operating expense per facility.

Two comments are warranted on the analysis for Objective 2. First, prior research suggested that NHs with higher quality (which might occur if the STM benchmark increased quality) had lower per resident operating expenditures (Mukamel & Spector, 2000; Weech-Maldonado et al., 2003). These efficiency gains could offset some of the increase in expenses associated with staffing to the STM benchmark. However, the size of this offset is unknown and likely to be small relative to the increased operational expense of hiring additional staff; therefore, it was not considered in this study. Second, while some NHs could slightly reduce operating expenses if staffed above the STM benchmark, this study treated their operating expenses as unchanged. It is infeasible for Medicare and Medicaid to claw back reductions in operating expenses if a NH reduced staffing levels to the STM benchmark. In this scenario, any operating expense savings could go towards nonnursing staff expenses or profits. There is also no guarantee that implementing an STM benchmark would result in any facility reducing staffing levels, as there could be multiple reasons for NHs to be staffed above the STM benchmark. Therefore, the calculations in this study best reflected the additional expenses that needs to be funded by decreased profits, reduced nonnursing staff expenses (e.g., ancillary services), or increased revenues.

## Results

**Figure 1.**
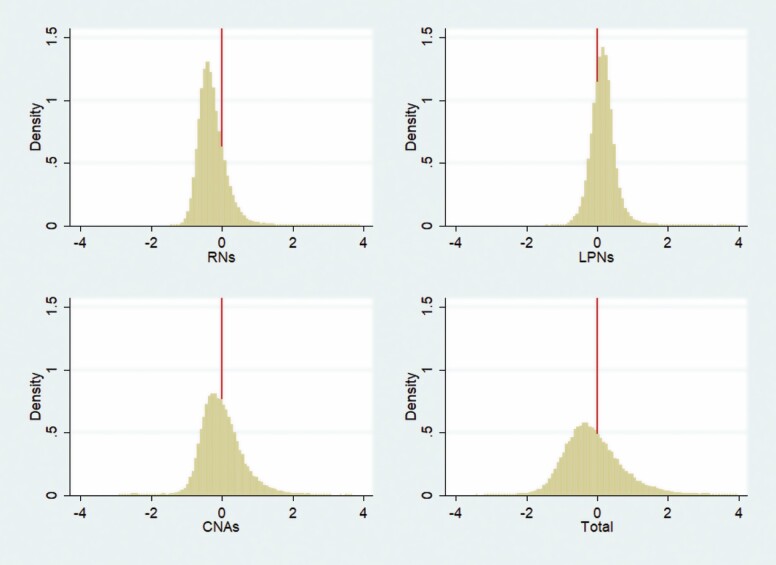
Histograms of the difference in actual to STM benchmark staffing levels among recertification surveys from January 1, 2014 to March 31, 2018. The figure reports a histogram of the difference in actual and STM benchmark staffing levels for each type of nursing staff and total nursing staff. The source of the data are recertification inspections that occurred between January 1, 2014 and March 31, 2018 in the continental United States among freestanding NHs certified before 2013. The sample size is 54,886. CNA = certified nurse aide; LPN = licensed practical nurse; NH = nursing home; RN = registered nurse; STM = Staff Time Measurement.

The results associated with Objective 1 indicated the majority of NHs had actual staffing levels below the STM benchmark. Histograms of the difference in actual and STM benchmark staffing levels (Figure 1) found that RNs, CNAs, and total nursing staffing were more likely to have negative differences (i.e., actual < STM benchmark). These are verified in Table 1. The proportion of recertification surveys with staffing levels below the STM benchmark was 80.2% for RNs, 54.4% for CNAs, 60.0% for total nursing staffing, and 28.4% for LPNs. When facilities were staffed below the STM benchmark, the amounts were not trivial, averaging 0.41 HPRD below the STM benchmark for RNs and 0.62 HRPD below the STM benchmark for total nursing staff. For reference, the mean actual RN and total staffing levels for all recertification surveys in the analytic sample were 0.77 and 4.04 HPRD, respectively. Regardless of whether the actual staffing level was above or below the STM benchmark, a nearly equal proportion of surveys resulted in a nursing staff deficiency. The frequency of “sufficient nursing staff” deficiencies issued was around 3% of recertification surveys and about 1% for “registered nurse” deficiencies.

**Table 1.** Comparison of Actual and STM Benchmark Nursing Staff Levels for Recertification Surveys and Corresponding Nursing Staff Deficiencies

Nursing staff type				Received a deficiency for	
	Actual staffing level ≥ STM benchmark	Recertification surveys (%)	Average difference in actual and STM benchmark staffing level (HPRD)	Sufficient nursing staff (%)	Registered nurses (%)
Registered nurses (RN)	Yes	19.84		2.83	0.47
	No	80.16	−0.41	2.85	1.03
Licensed practical nurses (LPN)	Yes	71.58		2.62	1.05
	No	28.42	−0.19	3.40	0.58
Certified nurse aides (CNA)	Yes	45.64		2.27	0.86
	No	54.36	−0.40	3.32	0.96
Total nursing staff (RN + LPN + CNA)	Yes	40.32		2.18	0.78
	No	59.68	−0.62	3.29	1.01

*Notes:* The table reports the proportion of recertification surveys in which actual nursing staff levels were above or below the STM benchmark, the difference between actual and STM benchmark staffing levels, and the proportion of recertification surveys that result in a nursing staff deficiency. Sufficient nursing staff deficiencies are identified with F-tags 353 and 725 and registered nurse deficiencies with F-tags 354 and 727. The source of the data is the Nursing Home Compare Archive data. The table reports information for recertification surveys completed from January 1, 2014 to March 31, 2018 that also report actual and STM benchmark staffing levels. The sample is restricted to freestanding nursing homes in the continental United States that were first certified in 2013. The sample size is 54,886. HPRD = hours per resident day; STM = Staff Time Measurement.

The first set of results for Objective 2, which quantifies the additional annual operating expenses associated with staffing to the STM benchmark in 2017, are reported in Table 2. Among all NHs in the analytic sample, 59.1% of NHs incurred additional operating expenses in order to staff to the STM benchmark. The mean and median additional expenses for these NHs were $538,090 and $388,178, respectively. For comparison, the mean and median net patient revenue in the analytic sample was $9.7 and $8.1 million per facility, respectively. These additional costs, at then prevailing reimbursement rates, resulted in 72.4% of NHs losing money caring for residents. Because some studies found that Medicaid reimbursed below the cost of care (Eljay, 2016), Table 2 also reports the results stratified by Medicaid payer-mix (i.e., Medicaid resident days divided by annual resident census from the MCRs―Worksheet S-3 Part I). As a NH’s Medicaid payer-mix increased, facilities were more likely to incur additional operating expenses, those operating expenses were larger, and the NH was more likely to be unprofitable.

**Table 2.** Additional Operating Expenses to Staff to the STM Benchmark and Profitability of Nursing Homes

Sample and subsamples	Sample size	Additional operating expenses to meet STM benchmark			% of unprofitable nursing homes
		Effected facilities (%)	Mean increase ($)	Median increase ($)	
Entire sample	12,117	59.08	538,090	388,178	72.39
Medicaid payer-mix (0%–50%)	3,842	44.61	478,221	337,578	71.14
Medicaid payer-mix (51%–75%)	5,406	62.26	531,184	376,683	71.09
Medicaid payer-mix (76%–100%)	2,869	72.46	598,630	450,811	76.16
No home office or related party transactions	2,604	47.89	564,731	380,902	77.00
Government-owned facilities	776	60.05	555,420	433,185	76.55

*Notes:* The table reports the percentage of nursing homes that would need to increase operating expenses to meet the STM benchmark, the mean and median of that increase, and the proportion of all nursing homes that are unprofitable staffing to the STM benchmark or at current staffing levels if staffing above the STM benchmark. The source of the data is the Nursing Home Compare Archive data and Medicare Cost Reports ending in Fiscal Year 2017. The sample is restricted to freestanding nursing homes in the continental United States opened for the full fiscal year that do not have outlier staffing levels or wage rates. These calculations are performed for the entire sample and subgroups of nursing homes. STM = Staff Time Measurement.

To understand the scope of the increased operating expense across states, Table 3 reports by state the proportion of NHs that would incur additional expenses to staff to the STM benchmark, and among the facilities that experienced an increased operating expense, the amount of those increases. The increased operating expenses were reported on an annual basis per resident and per facility, as well as the aggregate amount for the entire state. The increases in operating expenses were not equally dispersed across states. For example, 5.6% of NHs incurred additional operating expenses in Delaware at an average cost of $760 per resident year, or about $85,000 per NH (among the 5.6%). In contrast, 91.6% of Louisiana NHs incurred additional operating expenses at an average cost of $6,588 per resident year, or about $663,000 per NH (among the 91.6%). The top five states with the highest additional operating expenses per NH were New York ($1.67 million), the District of Columbia ($1.1 million), Illinois ($793,327), Texas ($731,490), and Pennsylvania ($674,444). In aggregate, the additional operating expenses for the facilities in the analytic sample that incurred additional operating expenses to staff to the STM benchmark would have needed to be increased by $3.8 billion dollars. Because the analytic sample included only a subset of NHs, identifying the total number of freestanding NHs from the NHC Archive data for 2017 and assuming each state’s additional operating expenses would increase in the same proportion, the national estimate increased to $4.9 billion for 2017.

**Table 3.** Additional Operating Expenses to Staff to Staff Time Measurement (STM) Benchmarks Among Nursing Homes Requiring Additional Expenses

State	Nursing homes with increased expenses (%)	Annual increased operating expenses			State per facility operating expense ranking
		Per resident ($)	Per facility ($)	State aggregate ($)	
Alabama	41.2	2,306	241,207	17,608,130	38
Arkansas	45.0	2,572	202,572	17,218,632	41
Arizona	42.9	4,198	391,676	19,975,493	20
California	68.3	5,878	538,562	360,297,752	12
Colorado	42.3	4,709	401,709	32,136,743	19
Connecticut	35.7	3,378	357,249	25,364,690	25
District of Columbia	22.2	4,168	1,084,485	2,168,969	2
Delaware	5.6	760	85,224	170,449	49
Florida	33.4	3,034	341,089	70,946,454	28
Georgia	89.4	6,499	624,019	152,260,745	9
Iowa	55.3	3,247	169,825	35,323,598	44
Idaho	19.3	4,778	252,691	2,779,605	36
Illinois	61.4	7,885	793,327	259,417,803	3
Indiana	71.6	6,667	491,166	177,311,003	15
Kansas	39.6	3,667	202,252	18,000,450	42
Kentucky	72.7	4,851	432,604	77,003,593	16
Louisiana	91.6	6,558	663,313	137,969,129	7
Massachusetts	23.7	3,397	364,985	29,198,824	24
Maryland	48.7	4,901	579,679	55,649,203	11
Maine	19.0	3,156	168,592	2,023,098	45
Michigan	40.4	4,011	350,626	52,243,317	27
Minnesota	13.0	1,721	107,622	3,551,523	48
Missouri	66.5	3,347	266,004	69,693,062	34
Mississippi	66.3	3,290	282,875	29,984,801	32
Montana	55.3	3,769	227,980	5,927,474	40
North Carolina	77.1	7,109	666,504	181,289,029	6
North Dakota	17.6	2,390	129,891	779,345	47
Nebraska	39.5	3,495	166,662	9,666,391	46
New Hampshire	40.7	3,454	354,303	7,794,663	26
New Jersey	51.3	4,745	632,005	99,224,735	8
New Mexico	72.7	3,419	305,630	12,225,193	30
Nevada	59.0	4,597	493,931	11,360,409	14
New York	68.4	10,290	1,665,837	408,130,017	1
Ohio	77.9	6,256	500,480	331,818,034	13
Oklahoma	68.4	3,523	235,803	28,532,215	39
Oregon	35.8	3,829	245,604	9,578,543	37
Pennsylvania	60.9	5,922	674,444	232,008,892	5
Rhode Island	42.0	3,571	386,516	11,208,961	22
South Carolina	60.9	4,008	411,139	39,058,191	18
South Dakota	54.3	3,447	190,976	7,257,106	43
Tennessee	60.5	4,530	390,363	55,041,171	21
Texas	87.2	8,923	731,490	557,395,180	4
Utah	62.9	4,688	298,343	13,127,077	31
Virginia	65.6	5,357	587,752	87,575,061	10
Vermont	23.1	3,245	276,471	1,658,824	33
Washington	42.3	5,046	421,665	29,094,885	17
Wisconsin	40.9	4,109	265,832	31,634,037	35
West Virginia	83.1	4,512	384,407	28,446,123	23
Wyoming	60.0	4,939	338,294	4,059,523	29
Continental United States	59.1	5,905	538,111	3,852,188,145	

*Notes:* The table reports the proportion of nursing homes that need to increase operating expenses to meet the STM benchmark, and among these nursing homes, the annual increase in operating expenses the nursing home would need to incur on a per resident and per facility basis, and aggregated to the entire state. The final column reports the state ranking for increased operating expenses on a per facility basis (1 = highest; 49 = lowest). The source of the data is the Medicare Cost Reports ending in Fiscal Year 2017 matched with STM benchmark staffing levels obtained from the Nursing Home Compare Archive data. The sample is restricted to freestanding nursing homes in the continental United States opened for the full fiscal year that do not have outlier staffing levels or wage rates.

## Discussion and Implications

The coronavirus (COVID-19) pandemic has brought systemic concerns regarding NH quality to the forefront of national attention. Early in the pandemic, COVID-19 cases and deaths were concentrated among NH residents, and throughout the pandemic, the industry reported shortages of nursing staff (McGarry et al., 2020; Xu et al., 2020). With insights from the pandemic, improving NH quality in the future requires a closer look at how NHs are staffed. Numerous studies have examined the association with NH quality and nursing staff levels, with one study questioning the quality of the literature (Armijo-Olivo et al., 2020). While the relationship is complex and multiple studies find inconclusive evidence (Backhaus et al., 2014; Harrington et al., 2012), the consensus is that higher nursing staff levels are generally associated with higher quality (Bowblis, 2011; Chen & Grabowski, 2015; Park & Stearns, 2009; Tong, 2011).

NH regulations require facilities to have sufficient nursing staff levels to care for residents, but currently, federal regulations do not clearly define the staffing level required to be sufficiently staffed. Given this lack of a CMS benchmark, NHs rely on a community standard of care, which some advocates argue does not provide adequate staffing levels to care for residents. A recent recommendation by Harrington et al. (2020) is for CMS to construct a quantitative measure of resident acuity that acts as a benchmark to determine whether a NH is sufficiently staffed. Using RUG billing codes from the MDS, and nursing staff times associated with RUG billing code from the STM studies, Harrington and colleagues outlined a way to calculate an STM benchmark. The authors argue that the STM benchmark is likely associated with being sufficiently staffed, would increase staffing levels from current levels, and would potentially increase quality. However, the STM benchmark has not been empirically tested. It is unknown whether the benchmark has any relationship to how regulators currently assess whether a NH is sufficiently staffed, if it is associated with higher quality or being sufficiently staffed, or if the STM benchmark is economically feasible.

In empirically testing this methodology, this study found the vast majority of NHs did not staff to the STM benchmark. Over 4 out of every 5 recertification surveys that occurred from 2014 to March 2018 were found to have RN staffing levels below the STM benchmark. Over half and 3 out of every 5 recertification surveys resulted in the facility staffing below the STM benchmark for CNAs and total nursing staffing, respectively. Not only were NHs not using the STM benchmark to determine their nursing staff levels, NH regulators were also not using the STM benchmark to determine if a NH is sufficiently staffed. If regulators were using the STM benchmark to determine regulatory compliance, the vast majority of NHs that failed to staff to the STM benchmark would have received a nursing staff deficiency. Additionally, there would be no nursing staff deficiencies issued to NHs that staffed above the STM benchmark. Even though the majority of recertification surveys reported staffing levels below the STM benchmark, no more than about 3% of these surveys had an associated deficiency issued for “sufficient nursing staff” and no more than about 1% had a deficiency for “registered nurses.” Interestingly, NHs staffed above the STM benchmark also received nursing staff deficiencies.

While CMS, state regulators, and NHs were not and currently do not use the STM benchmark to determine sufficient nursing staff levels, a key finding of this paper is the STM benchmark would likely increase the overall staffing levels of many NHs if it eventually became a regulatory requirement. This could lead to an improvement in a number of quality measures, assuming that NHs do not offset the nurses they need to hire by decreasing staffing of ancillary services, something research has found when states increase minimum nursing staff requirements (Bowblis & Hyer, 2013; Thomas et al., 2010). Ancillary services, such as social service or activities staff, are important for the quality of life and care. Past research has shown social services, and activities staff can be more cost-effective at improving some dimensions of quality than nursing staff (Bowblis & Roberts, 2020). While this study does not examine whether staffing to the STM benchmark would result in improved quality, any potential improvements gained by staffing to the STM benchmark would need to be counterbalanced with the economic costs of implementing such a benchmark.

Adopting the STM benchmark as a federal requirement would increase the operating expenditures for nearly 3 out of every 5 NHs in the United States in 2017. For NHs that needed to increase expenditures, the average increase in expenditures was over half a million dollars per facility (or about 5.5% of net patient revenues). For some NHs, this increase is likely to be paid out of profits. Nevertheless, on a national basis, the STM benchmark is not economically feasible for most NHs. The average profit margin of NHs is below 1% (Weech-Maldonado et al., 2019), and increased operating expenditures to reach the STM benchmark would cause a significant number of NHs to become unprofitable. Without being offset by additional revenue, implementing the STM benchmark would cause nearly three quarters of the NH industry to lose money providing services to residents and could result in the majority of NHs declaring bankruptcy or closing. Yet, not all states were affected equally. Increased operating expenses varied across states, from a low of about $85,000 to over $1.67 million annually per facility. States that had larger per NH increases in operating expenses were generally associated with higher nursing staff wage rates, lower per diem Medicaid reimbursement rates, and lower or no state regulations mandating a minimum nursing staff level.

Across the entire United States, the additional operating expense staffing to the STM benchmark was approximately $4.9 billion per year (see Author Note 6). While some of this expense is likely to be offset from NH profits, the low profit margins of NHs means that most of this increase in operating expense would have to either come from decreasing nonstaffing expenses (e.g., activities, food quality) or increasing revenues. Any revenue offset would need to come from the government as the greatest share of NH revenues come from the Medicare and Medicaid programs. The Medicaid program would face the largest financial burden. NHs with the greatest reliance on Medicaid-reimbursed residents would more likely need additional expenses and have larger additional expenses to staff to the STM benchmark. This means the political feasibility of implementing the STM benchmark depends on the willingness of federal and state lawmakers to increase taxpayer spending on NH care. This may be politically and economically impossible in states that have the largest gap between actual and STM benchmark staffing levels without federal assistance.

### Limitations

As with all studies, this study had limitations. First, Objective 1 utilized actual nursing staff levels calculated from data collected during recertification surveys (i.e., CMS-671 and CMS-672). Because the CMS-672 only reports the facility census for one day, while the CMS-671 reports staffing hours worked over a 14-day period, this can lead to measurement error in actual staffing levels. This measurement error is more likely to be acute if the facility’s census changed significantly from the 14-day pay period in which hours worked were obtained and census reported on the CMS-672. Given the large differences in actual and the STM benchmark staffing levels, this concern was unlikely to affect the conclusions drawn from the analysis. Furthermore, the NHC Archive data were the best source of publicly available information for NH nursing staff levels when surveyors were assessing whether the NH is substantially compliant with nursing staff regulations and when the STM benchmark was calculated by CMS.

Second, Objective 2 determined whether each NH was profitable after accounting for the additional operating expenses associated with the STM benchmark. A potential criticism levied against the NH industry is the use of complex corporate structures or services purchased from related parties to hide profits (Harrington et al., 2015). To address this potential criticism, Table 2 also reported results for two subsamples of NHs that were unlikely to be subject to these criticisms: (a) NHs with no home offices or related party payments, and (b) government-owned NHs. In the first case, there were no complex corporate arrangement or related parties. In the second case, government-owned organizations were not profit-motivated. Both of these subsamples came to qualitatively similar conclusions as examining all NHs in the analytic sample.

Third, the average wage rate paid by the NH was utilized to calculate the additional operating expense for each facility. This was likely to result in an underestimate of the actual expense of increasing staffing levels to the STM benchmark. First, it assumes that there was a willing and able workforce available for NHs to hire. There is currently a shortage of NHs workers (Stone, 2018) and hence NHs may not be able to find additional nursing staff. Second, related to the first, was if demand for nursing staff increases, the law of supply and demand predicts that wage rates would have needed to increase, especially in labor markets already experiencing workforce shortages. This would result in higher wages than the wage rates used in additional operating expense calculations.

Fourth, this study did not examine the underlying feasibility of using the STM benchmark. RUG billing codes are retrospective and require look-back periods, meaning RUG billing codes may not be available for some residents. This study had not assessed whether it is practicable for NHs to calculate and implement the STM benchmark, as it would require NHs to calculate RUG billing codes on a regular basis, anticipate changes in resident acuity, and require flexibility in staffing that may not be feasible. Furthermore, this study assumed that the RUG billing codes used by CMS are a reflection of a NH’s overall acuity level, and that RUG billing codes accurately measured all the needs and preferences of NH residents. This study had not assessed these assumptions. If this or similar acuity-based benchmarks are considered by policymakers in the future, additional research is warranted to determine the feasibility of its application in a real-world setting. This includes assessing the underlying assumptions of whether the mechanism used to measure acuity appropriately accounts for residents’ needs and preferences. It also includes assuring the nursing staff times associated with those measures of acuity are associated with providing appropriate care for NH residents.

And finally, this study did not assess whether staffing to the STM benchmark would have resulted in improved quality of care or quality of life. While many NHs would have needed to increase nursing staff levels, there is no guarantee setting a benchmark staffing level will result in improved quality for every NH and resident. Furthermore, the STM studies, in which the STM benchmark is based, did not account for changes in care practices, technology, or changes in nursing scope of practice laws over the last 25 years. The STM studies were also not designed to determine if the nursing staff times associated with RUG billing codes were “sufficient” (see Author Note 7). In summary, additional research is needed to assess whether staffing to the STM benchmark levels or any other benchmark would result in improved quality and is consistent with being sufficiently staffed.

## Conclusion

Without clear quantitative guidance on the staffing level needed to be sufficiently staffed, most NHs are subject to a community standard of care, which is determined by the market or via minimum nursing staff regulations dictated by the state. One proposed way to provide this guidance is to implement an acuity-based benchmark based on RUG billing codes and nursing staff times from the STM studies, as proposed by Harrington et al. (2020). Implementing this STM benchmark would cause most NHs to increase nursing staff levels, but at the same time would increase their operating expenses. These increased expenses need to be offset by increased reimbursement from Medicare and Medicaid, otherwise a significant number of NHs will lose money and close. While RUG billing codes are still calculated, Medicare has moved to the Patient Drive Payment Model, which acknowledged that RUG billing codes were primarily driven by reimbursement incentives. Moving forward, policymakers considering using minimum nursing staffing regulations, including acuity-based benchmarks similar to the one examined in this study, need to weigh the potential benefit of improved quality against the financial cost of paying for additional nursing staff. They must also consider whether the acuity measure used to construct the benchmark reflects the actual staffing needs of residents or is driven primarily by reimbursement incentives. This study provides a framework to determine the monetary cost associated with changes in minimum nursing staff regulations.

## Author Notes

1. The STM studies were conducted to help Centers for Medicare and Medicaid Services (CMS) determine reimbursement rates when the Prospective Payment System was rolled out in 1998. In addition, CMS utilized the Staff Time Measurement (STM) studies to calculate “case-mix hours,” which is a case-mix adjustment factor used to determine a nursing home (NH’s) staffing star ratings for the Five-Star Quality Rating System from 2009 until March of 2018 (CMS, 2018). The Staff Time and Resource Intensity Verification (STRIVE) study was used to update the Prospective Payment System by obtaining nursing staff times that accounted for changes in care practices since the STM studies. More details on the STM and STRIVE studies are available at Federal Register (1999, p. 41,663–41,665) and https://www.cms.gov/Medicare/Medicare-Fee-for-Service-Payment/SNFPPS/TimeStudy.

2. Harrington et al.’s (2020) primary proposal was to assign each Resource Utilization Group code nursing staffing times either from the STM studies or the “case-mix hours” used by CMS to determine a NH’s staffing star rating. Because CMS used the staff times from the STM studies to determine “case-mix hours” (see CMS, 2015, Appendix A), these two proposals were equivalent for the period of the inception of the Five-Star Quality Rating System through March 31, 2018.

3. CMS calculated and reported in the Nursing Home Compare (NHC) Archive data a measure of the STM benchmark as part of case-mix adjusting the staffing star ratings for the Five-Star Quality Rating System. In April 2018, CMS started to utilize the Payroll-Based Journaling data and stopped using the nursing staff times from the STM studies. Therefore, CMS’s calculated measures of the STM benchmark associated with each recertification survey are available from 2014 to March 2018.

4. The NHC Archive data obtained these staffing levels from the CMS-671, which reported the number of hours worked for each type of nursing staff during a 14-day pay period slightly before the recertification survey. The facility census was obtained from the CMS-672, which was also filled out during the recertification survey.

5. From the inception of the Five-Star Quality Rating System through March 2019, CMS’s documentation used the term “expected hours” to instead of “case-mix hours.” CMS changed to the term “case-mix hours” in April 2019 (CMS, 2019b). The term “case-mix hours” was used in this study because some stakeholders have misinterpreted CMS’s use of the term “expected hours” to imply that CMS “expected” or “required” these staffing times as a regulatory requirement. This is not supported by federal regulations. In fact, CMS (2017; §483.35(a)) clearly states there are no federal minimum nursing staff requirements.

6. The cost associated with implementing the STM benchmark would be even larger if nursing staff levels from the Payroll-Based Journaling data were utilized instead of Medicare Cost Reports.

7. The federal government cited the small sample size as a potential limitation of the STM studies stating, they “not believe that is appropriate to require staffing standards based on the needs of such a small proportion of the facility’s population” (Federal Register, 1999, p. 41,665).

## References

[CIT0001] Armijo-Olivo, S., Craig, R., Corbian, P., Guo, B., Souri, S., & Tjosvold, L. (2020). Nursing staffing time and care quality in long-term care facilities: A systematic review. Gerontologist, 60(3), e200–e217. doi:10.1093/geront/gnz053 31115444

[CIT0002] Backhaus, R., Verbeek, H., van Rossum, E., Capezuti, E., & Hamers, J. P. (2014). Nurse staffing impact on quality of care in nursing homes: A systematic review of longitudinal studies. Journal of the American Medical Directors Association, 5(6), 383–393. doi:10.1016/j.jamda.2013.12.080 24529872

[CIT0003] Bowblis, J. R . (2011). Staffing ratios and quality: An analysis of minimum direct care staffing requirements for nursing homes. Health Services Research, 46(5), 1495–1516. doi:10.1111/j.1475-6773.2011.01274.x 21609329 PMC3207189

[CIT0004] Bowblis, J. R., & Ghattas, A. (2017). The impact of minimum quality standard regulations on nursing home staffing, quality, and exit decisions. Review of Industrial Organization, 50(1), 43–68. doi:10.1007/s11151-016-9528-x

[CIT0005] Bowblis, J. R., & Hyer, K. (2013). Nursing home staffing requirements and input substitution: Effects on housekeeping, food service, and activities staff. Health Services Research, 48(4), 1539–1550. doi:10.1111/1475-6773.12046 23445455 PMC3725539

[CIT0006] Bowblis, J. R., & Roberts, A. R. (2020). Cost-effective adjustments to nursing home staffing to improve quality. Medical Care Research and Review, 77(3), 274–284. doi:10.1177/1077558718778081 29884092

[CIT0007] Centers for Medicare and Medicaid Services. (2015, February). Design for nursing home compare five-star quality rating system: Technical users’ Guide. Baltimore, MD: CMS.

[CIT0008] Centers for Medicare and Medicaid Services. (2017, November). State operations manual: Appendix pp- guidance to surveyors for long term care facilities. Baltimore, MD: CMS.

[CIT0009] Centers for Medicare and Medicaid Services. (2018, May). Design for nursing home compare five-star quality rating system: Technical users’ guide. Baltimore, MD: CMS.

[CIT0010] Centers for Medicare and Medicaid Services. (2019a, April). Payroll-based journal public use files: Technical specifications. Baltimore, MD: CMS.

[CIT0011] Centers for Medicare and Medicaid Services. (2019b, April). Design for nursing home compare five-star quality rating system: Technical users’ guide. Baltimore, MD: CMS.

[CIT0012] Chen, M. M., & Grabowski, D. C. (2015). Intended and unintended consequences of minimum staffing standards for nursing homes. Health Economics, 24(7), 822–8 39. doi:10.1002/hec.3063 24850410

[CIT0013] Eljay, L. L. C . (2016). A report on shortfalls in medicaid funding for nursing center care. American Health Care Association. https://www.ahcancal.org/Reimbursement/Medicaid/Documents/2017%20Shortfall%20Methodology%20Summary.pdf

[CIT0014] Federal Register. (1999). Medicare program; Prospective payment system and consolidated billing for skilled nursing facilities. 66(145), 41644–41683. 10558604

[CIT0015] Harrington, C., Dellefield, M. E., Halifax, E., Fleming, M. L., & Bakerjian, D. (2020). Appropriate nurse staffing levels for U.S. nursing homes. *Health Services Insights*, 13, 1–14. doi:10.1177/1178632920934785 PMC7328494 32655278

[CIT0016] Harrington, C., Olney, B., Carrillo, H., & Kang, T. (2012). Nurse staffing and deficiencies in the largest for-profit nursing home chains and chains owned by private equity companies. Health Services Research, 47(1 Pt 1), 106–1 28. doi:10.1111/j.1475-6773.2011.01311.x 22091627 PMC3447240

[CIT0017] Harrington, C., Ross, L., & Kang, T. (2015). Hidden owners, hidden profits, and poor nursing home care: A case study. International Journal of Health Services, 45(4), 779–800. doi:10.1177/0020731415594772 26159173

[CIT0018] McGarry, B. E., Grabowski, D. C., & Barnett, M. L. (2020). Severe staffing and personal protective equipment shortages faced by nursing homes during the COVID-19 pandemic. Health Affairs, 39(10), 1812–1821. doi:10.1377/hlthaff.2020.01269 32816600 PMC7598889

[CIT0019] Mueller, C., Arling, G., Kane, R., Bershadsky, J., Holland, D., & Joy, A. (2006). Nursing home staffing standards: Their relationship to nurse staffing levels. Gerontologist, 46(1), 74–80. doi:10.1093/geront/46.1.74 16452286

[CIT0020] Mukamel, D. D., & Spector, W. D. (2000). Nursing home costs and risk-adjusted outcome measures of quality. Medical Care, 38(1), 78–89. doi:10.1097/00005650-200001000-00009 10630722

[CIT0021] Park, J., & Stearns, S. C. (2009). Effects of state minimum staffing standards on nursing home staffing and quality of care. Health Services Research, 44(1), 56–78. doi:10.1111/j.1475-6773.2008.00906.x 18823448 PMC2669632

[CIT0022] Stone, R. I . (2018). Developing a quality direct care workforce: Searching for solutions. Public Policy and Aging Report, 27(3), 96–100. doi:10.1093/ppar/prx015

[CIT0023] Thomas, K. S., Hyer, K., Andel, R., & Weech-Maldonado, R. (2010). The unintended consequences of staffing mandates in Florida nursing homes: Impacts on indirect-care staff. Medical Care Research and Review, 67(5), 555–573. doi:10.1177/1077558709353325 20016032

[CIT0024] Tong, P. K . (2011). The effects of California minimum nurse staffing laws on nurse labor and patient mortality in skilled nursing facilities. Health Economics, 20, 802–8 16. doi:10.1002/hec.1638 20672247

[CIT0025] Weech-Maldonado, R., Lord, J., Pradhan, R., Davlyatov, G., Dayama, N., Gupta, S., & Hearld, L. (2019). High Medicaid nursing homes: Organizational and market factors associated with financial performance. Inquiry, 56, 1–9. doi:10.1177/0046958018825061 PMC6376504 30739512

[CIT0026] Weech-Maldonado, R., Neff, G., & Mor, V. (2003). Does quality of care lead to better financial performance?: The case of the nursing home industry. Health Care Management Review, 28(3), 201–2 16. doi:10.1097/00004010-200307000-00002 12940343

[CIT0027] Xu, H., Intrator, O., & Bowblis, J. R. (2020). Shortages of staff in nursing homes during the COVID-19 pandemic: What are the driving factors? JAMDA, 21, 1371–1377. doi:10.1016/j.jamda.2020.08.002 32981663 PMC7418696

